# Supplementary material for: Causal Relationship between Aging and Anorexia Nervosa: A White-Matter-Microstructure-Mediated Mendelian Randomization Analysis
Source: Biomedicines. 2024 Aug 16;12(8):1874. doi: 10.3390/biomedicines12081874 (PMC11351342; doi:10.3390/biomedicines12081874)
Supplement: Supplementary file 1 [file biomedicines-12-01874-s001.zip › Supplementary Figure/Figure S8/Leaveoneout plot of MR analysis for TL to ubm-b-2056.pdf]

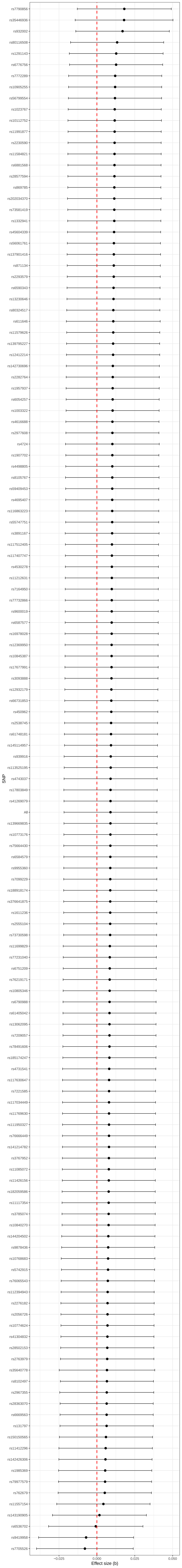
rs7790856
rs35446936
rs932002
rs80116508
rs1291143
rs6776756
rs7772289
rs10905255
rs56799554
rs1023767
rs10112752
rs11991877
rs2230590
rs11584821
rs6881568
rs28577594
rs869785
rs202034370
rs73581419
rs1332941
rs45604339
rs56061761
rs137901416
rs871134
rs2293579
rs6590343
rs13230646
rs80324517
rs611646
rs11579626
rs139795227
rs12412214
rs142730696
rs2282764
rs1957937
rs6054257
rs1003322
rs4616688
rs2977608
rs4724
rs1907702
rs4498805
rs8105767
rs59409453
rs4695407
rs116863223
rs55747751
rs3891167
rs117512405
rs117407747
rs4530278
rs11212631
rs7164950
rs77732866
rs9600019
rs6587577
rs16978028
rs12369950
rs10845387
rs17677991
rs3093888
rs12932179
rs66731853
rs450962
rs2538745
rs61748181
rs145114957
rs939916
rs113525195
rs4743037
rs17803849
rs41269079
All
rs139669835
rs10773176
rs75664430
rs6584579
rs9955360
rs7099229
rs188918174
rs376641875
rs1611236
rs2555104
rs73730598
rs11699829
rs77231040
rs6751209
rs76219171
rs10805346
rs6790988
rs61405042
rs13062095
rs7209057
rs78491606
rs185174247
rs4731541
rs117630647
rs7221585
rs117034449
rs11769630
rs111950327
rs76666449
rs141214782
rs3767952
rs11085072
rs11426156
rs182059586
rs11117354
rs3785074
rs10840270
rs144204502
rs9878436
rs10768683
rs5742915
rs76065543
rs112394943
rs2276182
rs2056726
rs10774624
rs41304832
rs28502153
rs2763979
rs35640778
rs8102497
rs2967355
rs28363070
rs6669563
rs131797
rs150150565
rs11412296
rs142426306
rs1985369
rs79977579
rs762679
rs11557154
rs143190905
rs6536702
rs9419958
rs7705526
SNP
-0.025
0.000
0.025
0.050
Effect size (b)
